# Correlation analysis of serum thyroid stimulating hormone with acute cerebrovascular disease

**DOI:** 10.1186/s40001-019-0395-4

**Published:** 2019-10-24

**Authors:** Jian Zhu, Ming Chen, Nan Li, Shaoling Yang, Lu Xu, Yanru Wang, Hong Li

**Affiliations:** 1 Department of Neurology, Dachang Hospital of Baoshan District, Shanghai, 200444 China; 2 Department of Endocrinology and Metabolism, Shanghai Tenth People’s Hospital, Tongji University School of Medicine, No. 301, Middle Yan Chang Road, Shanghai, 200072 China

**Keywords:** Acute cerebrovascular disease, Thyroid stimulating hormone, Cerebral infarction, Cerebral hemorrhage, Severity, Prognosis

## Abstract

**Background:**

Acute cerebrovascular disease (ACVD) could cause abnormal metabolism of thyroid hormones (TH), mostly represented as a euthyroid sick syndrome or low T3 syndrome. However, the changes in serum thyroid-stimulating hormone (TSH) are controversial. The aim of this study is to investigate the clinical significance of TSH alteration in patients with ACVD.

**Method:**

Patients with ACVD admitted in our hospitals between January 2013 and September 2017 were enrolled in this study (*n* = 245, including 176 cerebral infarctions and 69 cerebral hemorrhages). Their thyroid hormones were measured and compared with healthy individuals (*n* = 75). The correlation of TSH with severity and prognosis of ACVD were analyzed by receiver operating characteristic curve.

**Results:**

Serum TSH in ACVD group was higher than the control group (1.64 ± 1.08 vs. 1.26 ± 0.36 μIU/mL, *P* < 0.05). The TSH levels in intermediate and severe patients with ACVD were higher than in mild patients (1.72 ± 1.18 vs. 2.71 ± 0.93 vs. 1.02 ± 0.47 μIU/mL, *P* < 0.05). Receiver Operating Characteristic curve (ROC) of TSH in determining the severity of patients were 0.863 (Area under the curve, AUC), 1.496 μIU/L (optimal threshold), 76.5% (sensitivity) and 87.3% (specificity). TSH levels in improved and unchanged groups were significantly higher than the primarily healing group (2.27 ± 1.11 vs. 2.88 ± 1.07 vs. 0.86 ± 0.46 μIU/mL, *P* < 0.05). ROC of TSH in determining the prognosis of patients was 0.910 (AUC), 1.681 mIU/L (optimal threshold), 79.8% (sensitivity) and 90.5% (specificity) correspondingly.

**Conclusion:**

Since elevated TSH in ACVD patients affects the outcome of thyroid function evaluation, it is preferable to re-check after the acute period. A correlation between a high TSH level and the severity and prognosis of ACVD was detected, but the mechanism of this correlation needs to be further studied.

## Introduction

Cerebrovascular disease (CVD) is a common and frequently-occurring nervous system, which accounts for about 10% of all diseases, and is one of the three leading causes of human diseases. Acute cerebrovascular disease (ACVD), because of its disability rate and high mortality, seriously affects the quality of life of the patients with the disease, bringing a heavy financial burden to the family and society. ACVD has many aspects of damage and influence on human body, which can not only cause the damage of nervous system function, but also can cause a series of changes in endocrine and metabolism. The pathological basis of ACVD can be caused by cerebral thrombosis, result from ischemic cerebral infarction, or can be rupture of blood vessel in brain leading to cerebral apoplexy, this kind of disease also called a stroke.

A number of studies in recent years have discovered that ACVD and many other severe non-thyroid diseases could cause abnormal metabolism of thyroid hormones (TH) [1–4], mostly represented as a euthyroid sick syndrome (ESS) or low T3 syndrome. However, the results of research on changes in serum thyroid-stimulating hormone (TSH) are controversial or even conflicting [5, 6]. TSH is the most sensitive index for assessing the thyroid function and its alteration may directly affect the outcome of assessment on the thyroid function. Therefore, the aim of the present study was to define the degree of ACVD impact on TSH level by observing serum TSH changes in ACVD patients, and analyze the correlation between serum TSH change and the degree of severity of ACVD patients, in an attempt to provide experimental references for accurate assessment of the thyroid function.

## Materials and methods

### Subjects

Written informed consent was obtained from patients under a protocol approved by the Ethics Committee of Shanghai Dachang Hospital of Baoshan District. Included in this study were 245 ACVD patients who were admitted in Dachang Hospital between January 2013 and September 2017. The diagnosis of ACVD was in accordance with the diagnosis criteria specified in the Chinese Guidelines for Prevention and Treatment of Cerebrovascular Disease [7] and confirmed by head CT scan. All patients were hospitalized for more than 14 days except deaths. The exclusion criteria were: (1) patients with thyroid and adrenal gland diseases as confirmed by medical history inquiry and B-type ultrasound; (2) patients on long-term administration of drugs such as iodine-based and anti-thyroid drugs that may affect the thyroid function; and (3) patients with severe liver and kidney dysfunction or tumors. During physical examination, additional 75 healthy individuals without a thyroid, adrenal gland, heart, liver, brain, and kidney diseases were selected from the physical examination center of the hospitals and used as control.

### Grouping

Among the 245 ACVD patients, 176 patients were inflicted with acute ischemia cerebral vessels disease (AICVD), including 90 males and 86 females with a mean age of 75.03 ± 7.25 years, and the other 69 patients suffered from acute hemorrhagic cerebrovascular disease (AHCVD), including 39 males and 30 females with a mean age of 73.44 ± 8.64 years.

According to the American National Institutes of Health Stroke Scale (NIHSS), the 245 ACVD patients were graded and classified into three groups: the mild ACVD (mACVD) group (score: ≤ 6, *n* = 118), the intermediate ACVD (iACVD) group (score: 7–14, *n *= 63), and the severe ACVD (sACVD) group (score: ≥ 15, *n *= 64).

The prognosis was assessed according to the scoring criteria of the clinical neurological function deficit degree of stroke patients [8] by using the following equation: (admission NIHSS score subtract NIHSS score on day 14 after admission)/admission NIHSS score * 100%. Patients with a score of 91–100% fell in the basically cured group (*n *= 27); patients with a score of 46–90% fell in the significantly improved group (*n *= 120); patients with a score of 18–45% fell in the improved group (*n *= 59); patients with a score < 17% fell in the no change group (*n *= 24), and patients with an unchanged score + death fell in the deterioration group (*n *= 15). As the deterioration group included dead patients, it was unable to assess the NIHSS score on day 14 after admission, in addition, most patients in this group developed severe complications that may interfere with TSH and other THs outcomes. For these reasons, they were not included for prognosis comparison. The 75 healthy individuals in the control group included 40 males and 35 females with a mean age of 73.52 ± 6.45 years.

### Detection methods

Serum TSH, TT3, TT4, FT3 and FT4 were measured with an automatic chemiluminescence immunoassay analyzer (ADVIA Centaur, Siemens) and appropriate reagents. The reference values of these indexes are as follows: TSH (0.300–5.200 μIU/mL), TT3 (0.9–2.8 nmol/L), TT4 (65.6–179.2 nmol/L), FT3 (3.5–6.5 pmol/L), and FT4 (11.9–26.0 pmol/L).

### Statistical analysis

Data are expressed as mean ± SD. TSH, TT3, TT4, FT4, and FT3 were statistically analyzed by using the SPSS20.0 software. The inter-group comparison was performed by *t* test, and multi-group comparison was performed by analysis of variance. *P* values < 0.05 were considered statistically significant.

## Results

### General data of the included patients and healthy controls

There were no significant differences in age, M/F ratio, systolic blood pressure (SBP), diastolic blood pressure (DBP), serum creatinine (CR), blood urea nitrogen (BUN), alanine aminotransferase (ALT), and aspartate aminotransferase (AST) among the cerebral infarction, cerebral hemorrhage and healthy control groups (*P* > 0.05), indicating that they were comparable (Table 1).

**Table 1:** Comparison of the general data between cerebral infarction, cerebral hemorrhage and healthy control groups

Variables	Healthy control	Cerebral hemorrhage	Cerebral infarction	Normal value
Age (years)	73.52 ± 6.45	73.44 ± 8.64	75.03 ± 7.25	
Sex (M/F)	40/35	39/30	90/86	
SBP (mmHg)	130.6 ± 17.3	142.3 ± 22.5	135.9 ± 13.5	90.0–140.0
DBP (mmHg)	88.1 ± 15.8	85.0 ± 14.9	80.8 ± 9.6	60.0–90.0
CR (Umol/L)	61.9 ± 6.8	73.6 ± 15.7	68.3 ± 13.7	44.0–88.0
BUN (mmol/L)	5.6 ± 1.2	5.8 ± 1.8	5.7 ± 1.3	1.7–8.3
ALT (U/L)	22.8 ± 8.9	22.6 ± 12.3	24.1 ± 10.8	0–60.0
AST (U/L)	19.9 ± 5.2	22.8 ± 10.2	21.6 ± 6.0	0–38.0

*SBP* systolic blood pressure, *DBP* diastolic blood pressure, *CR* serum creatinine, *BUN* blood urea nitrogen, *ALT* alanine aminotransferase, *AST* aspartate aminotransferase

### Comparison of serum TH between the AVCD group and healthy control group

**Fig. 1:**
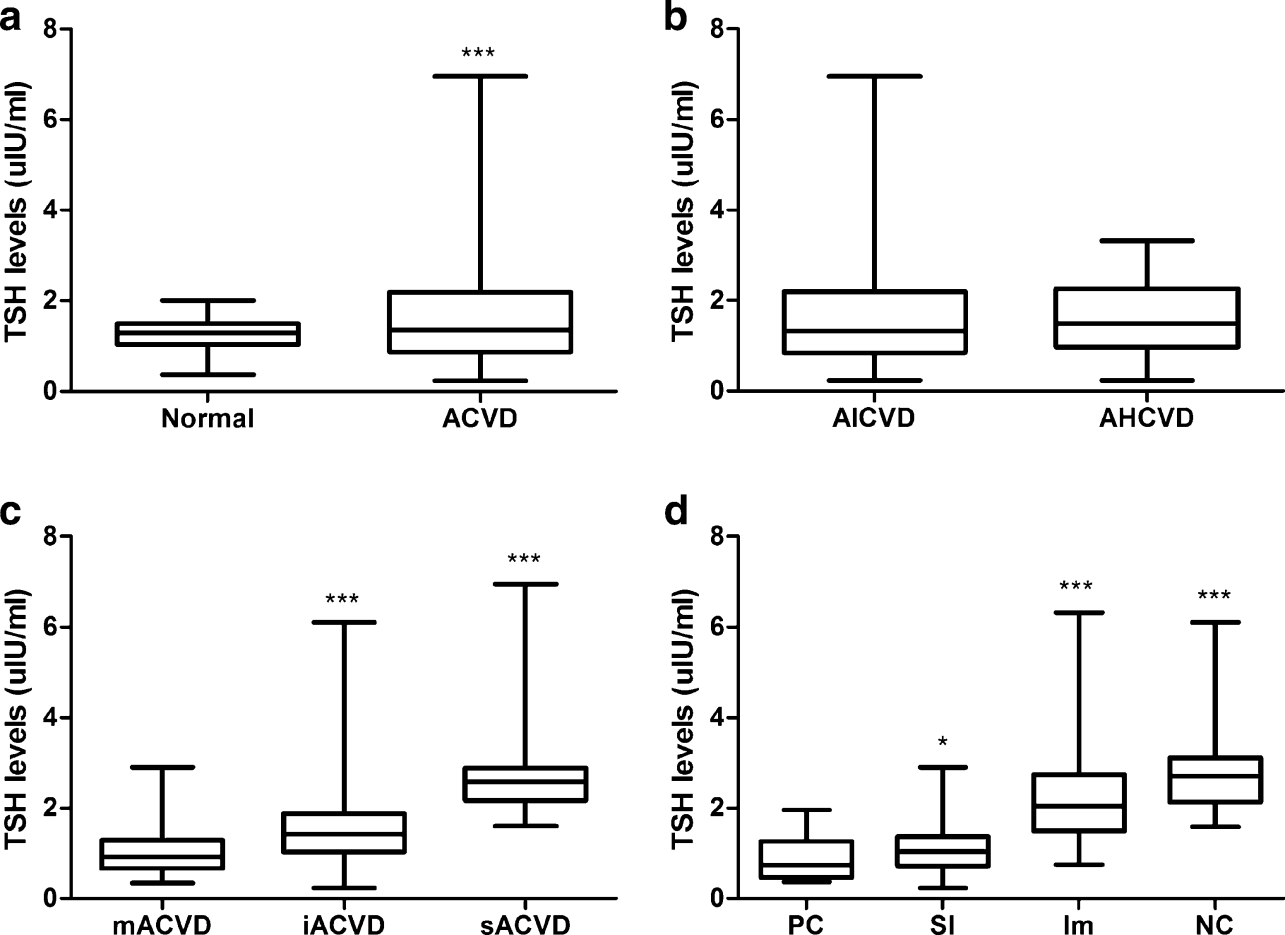
The levels of TSH in different groups. **a** The levels of TSH were higher in the AVCD group than that in the normal group. **b** There was no significant difference in TSH levels between AIVCD and AHCVD group. **c** The levels of TSH were increased in iACVD and sACVD group, compared with mACVD group. **d** The levels of TSH were increased in SI, Im and NC group, compared with PC group. *P < 0.05, ***P < 0.001

Compared with the healthy control group, serum TT3, FT3, FT4 were decreased and TSH was increased in ACVD group (both *P* < 0.05), while there was no significant difference in the level of TT4 (*P* > 0.05) (Table 2 and Fig. 1a).

**Table 2:** Comparison of serum TH between AVCD group and healthy control group

Group	Cases (*n*)	TT3 (nmol/L)	TT4 (nmol/L)	FT3 (pmol/L)	FT4 (pmol/L)	TSH (μIU/mL)
AVCD	245	1.32 ± 0.41*	113.00 ± 26.89	4.01 ± 0.85*	16.98 ± 3.20*	1.64 ± 1.08*
Normal	75	1.67 ± 0.25	105.86 ± 18.11	4.39 ± 0.47	18.41 ± 3.89	1.26 ± 0.36

* *P* < 0.05, compared with normal group

### Comparison of TH between AICVD and AHCVD groups

There was no significant difference in serum TT3, FT3, TT4, FT4, and TSH between AICVD and AHCVD groups (all *P* > 0.05) (Table 3 and Fig. 1b).

**Table 3:** Comparison of serum TH between AICVD group and AHCVD group

Group	Cases (*n*)	TT3 (nmol/L)	TT4 (nmol/L)	FT3 (pmol/L)	FT4 (pmol/L)	TSH (μIU/mL)
AICVD	176	1.33 ± 0.42	113.27 ± 26.84	4.08 ± 0.87	16.91 ± 3.02	1.64 ± 1.17
AHCVD	69	1.30 ± 0.40	112.32 ± 27.21	3.82 ± 0.78	17.15 ± 3.62	1.64 ± 0.80

### Comparison of TH between different severity groups

Compared with mACVD group, FT3 levels were decreased and TSH levels were increased in iACVD and sACVD groups (both *P* < 0.05), TT3 and FT4 were decreased only in sACVD group (*P* < 0.05). Compared with iACVD group, TT3, FT3, and FT4 were decreased and TSH was increased in sACVD group (both *P* < 0.05), whereas there was no significant difference in TT4 (*P* > 0.05) (Table 4 and Fig. 1c).

**Table 4:** Comparison of TH between different severity groups

Severity groups	Cases (*n*)	TT3 (nmol/L)	TT4 (nmol/L)	FT3 (pmol/L)	FT4 (pmol/L)	TSH (μIU/mL)
mACVD	118	1.50 ± 0.27	111.91 ± 24.75	4.47 ± 0.63	16.95 ± 2.88	1.02 ± 0.47
iACVD	63	1.42 ± 0.41	120.41 ± 25.61	4.00 ± 0.72*	17.99 ± 3.60	1.72 ± 1.18*
sACVD	64	0.89 ± 0.32*^#^	107.73 ± 30.54	3.16 ± 0.67*^#^	16.02 ± 3.09*^#^	2.71 ± 0.93*^#^

* *P* < 0.05, compared with mACVD group

^#^*P* < 0.05, compared with iACVD group

### TSH AUC analysis for judging the severity of ACVD

**Fig. 2:**
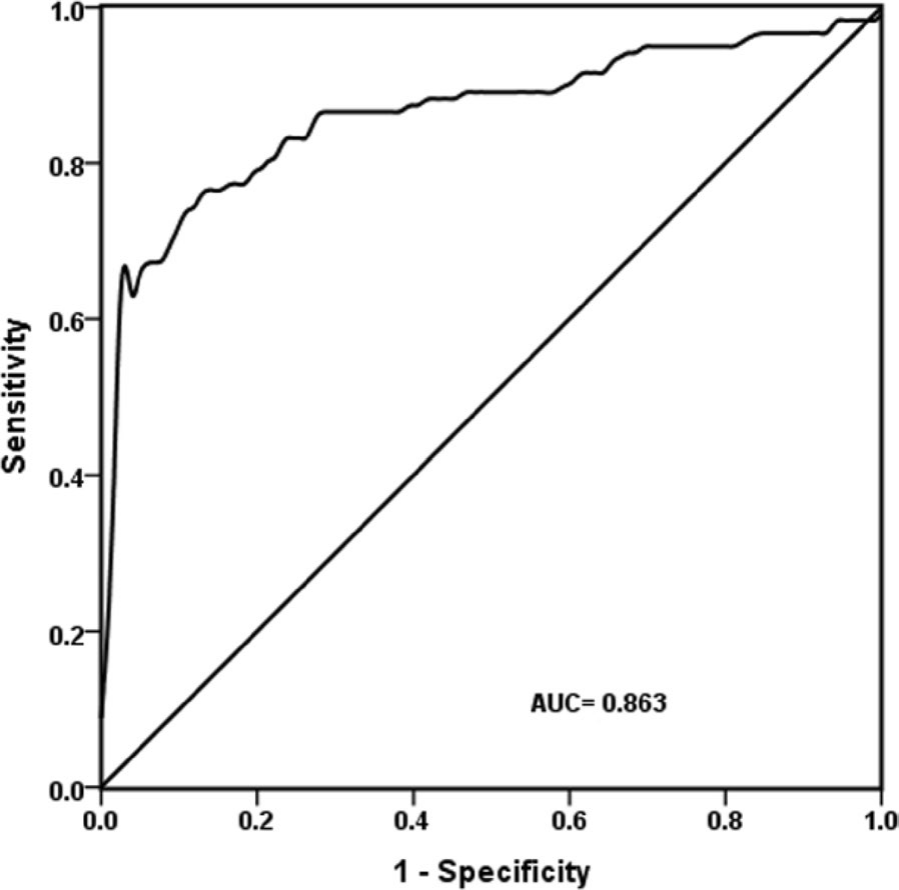
ROC curve plot for the severity of ACVD judged by TSH. The area under the ROC curve (AUC) of TSH for judging the severity of ACVD was 0.863

TSH AUC for judging the severity of ACVD was 0.863 (*P* < 0.01), the optimal threshold was 1.496 μIU/mL, sensitivity was 76.5%, and specificity was 87.3%, indicating that TSH level was closely correlated with the severity of ACVD (Fig. 2).

### Comparison of TH between groups with different prognosis

Compared with the PC group, TSH levels in rest all groups were increased (both *P* < 0.05), and TT3 and FT3 levels were decreased in Im and NC groups (both *P* < 0.05). Similarly, compared with SI group, TT3, and FT3 levels were decreased in Im and NC groups (both *P* < 0.05), and compared with Im group, FT3 levels were decreased in NC group (Table 5 and Fig. 1d).

**Table 5:** Comparison of TH between groups with different prognosis

Group	Case (*n*)	TT3 (nmol/L)	TT4 (nmol/L)	FT3 (pmol/L)	FT4 (pmol/L)	TSH (μIU/mL)
PC	27	1.51 ± 0.30	109.42 ± 26.64	4.51 ± 0.59	17.26 ± 3.03	0.86 ± 0.46
SI	120	1.47 ± 0.31	113.30 ± 24.45	4.34 ± 0.70	16.99 ± 2.91	1.10 ± 0.50*
Im	59	1.18 ± 0.48*^#^	116.13 ± 29.95	3.70 ± 0.75*^#^	17.32 ± 3.57	2.27 ± 1.11*^#^
NC	24	1.01 ± 0.32*^#^	120.30 ± 26.89	3.20 ± 0.57*^#∆^	16.48 ± 4.06	2.88 ± 1.07*^#∆^

* *P* < 0.05, compared with PC group

^#^*P* < 0.05, compared with SI group

^∆^*P* < 0.05, compared with Im group

### TSH AUC curve analysis for judging the prognosis of ACVD

**Fig. 3:**
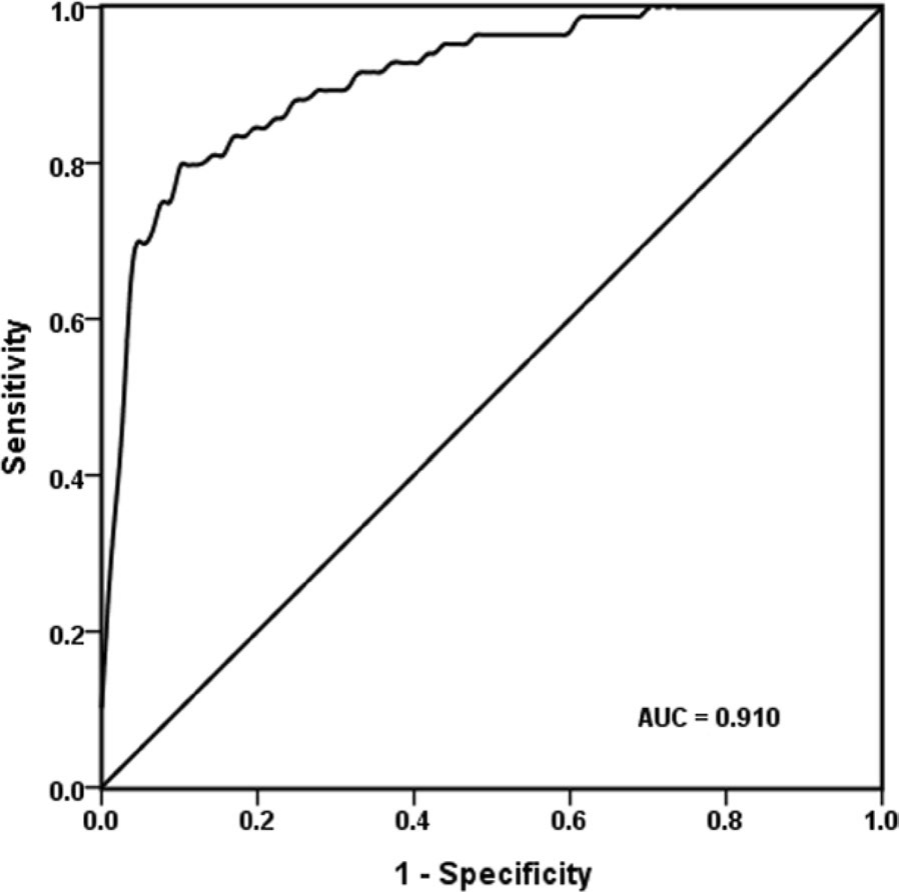
ROC curve plot for the prognosis of ACVD judged by TSH. The AUC of ROC analysis for TSH was 0.910 for judging the prognosis of ACVD

The TSH ROC curve for judging the prognosis of ACVD was 0.910 (*P* < 0.01), the optimal threshold was 1.681 μIU/mL, sensitivity was 79.8%, and specificity was 90.5%, indicating that TSH level was to some extent correlated with the prognosis of ACVD (Fig. 3).

## Discussion

TSH, a secretary hormone of adenohypophysis and a glycoprotein of 28–30 kDa, plays important roles in promoting the release and synthesis of TH, enhancing peroxidase activity and promoting thyroglobulin synthesis and tyrosine iodization. It is accepted as the most important and sensitive index for judging the thyroid function. According to the classical theory, TSH is regulated by thyrotropin-releasing hormone (TRH), T3 and T4. However, a number of studies have demonstrated that TSH is regulated and interfered by a variety of factors other than the hypothalamus–hypophysis–thyroid axis, and these influencing factors may mislead the correct assessment of the thyroid function [9]. ACVD is a neurological emergency. It can not only affect the brain function significantly but induce neuroendocrine abnormalities [10–12]. Current studies maintain that TH change during the acute stage of cerebrovascular disease (CVD) is mostly represented by ESS or low T3 syndrome characterized by a decrease in T3 and/or FT3. Their effect on TSH remains uncertain.

In the present study, it was found that the more severe the ACVD (the higher the NHISS score on admission), the more obvious the elevation trend of TSH. Serum TSH was higher than the reference value of 5.2 μIU/mL in patients with ACVD, and these patients were all in iACVD and sACVD groups, even though we found that the level of TSH in these two groups was no difference, at least indicating the amplitude of TSH change during the acute stage of CVD is correlated with the severity of the disease. In addition, patients with more pronounced elevation of TSH usually had a poorer clinical prognosis later two weeks after treatment, indicating that the severity of the condition is closely correlated with the prognosis and that the higher the TSH level on admission, the poorer the prognosis, which is consistent with the findings of other studies [13]. We postulate that ACVD is an important reason for the high serum level of TSH. The mechanism of causing ACVD-induced elevation of TSH may be explained by the following reasons. (1) Various ACVDs can induce changes in various endocrine hormones during the acute stage, such as changes in the RAS system and cortisol-ACTH axis [14]. The central regulation of thyroid function is impaired in patients with acute space occupying ischaemic stroke. These hormones may participate in TSH regulation. (2) Evidence indicated that inflammatory factors, such as IL-1b, soluble IL-2 receptor, IL-6, and tumor necrosis factor-α, have association with serum TSH levels [15, 16]. Large amounts of inflammatory factors released during the onset of acute cardiocerebrovascular diseases may participate in TSH regulation [17]. (3) Increased intracranial pressure or cerebral circulation dysfunction due to cerebral edema during acute stroke may cause abnormal pituitary hormone secretion, leading to abnormal TSH secretion [18]. (4) The hypothalamus–pituitary–thyroid axis determines all steps of TH biosynthesis and secretion [19]. When direct lesions of hypothalamus and pituitary such as degenerative necrosis of pituitary cells occur in ACVD patients, hormones secreted or stored in pituitary cells will release into the blood, resulting in the elevation of TSH [20]. Whether these changes will produce transient or permanent injury needs to be followed up and monitored.

In our present study, several limitations should be considered. The sample size is limited. The serum TSH levels were evaluated at a single time point. The patients included in this study were older, and the results were used to infer that the whole population of ACVD patients may be biased, which needs to be solved by further expanding the sample size and including more younger patients in the future.

## Conclusion

In summary, the present study demonstrated that ACVD could induce varying degrees of TSH elevation, and therefore clinicians should be alert to the misjudgment of the impact of transient elevation of TSH on the thyroid function. Improper use of Euthyrox based on the arbitrary judgment of hypothyroidism or subclinical hypothyroidism is a hidden danger to CVD patients, especially old-aged populations. To make an accurate assessment of the thyroid function, follow-up monitoring of TSH after the acute stage of CVD is recommended.

## Data Availability

All data generated or analyzed during this study are included in this published article.

## Abbreviations

ACVD: acute cerebrovascular disease
AICVD: acute ischemia cerebral vessels disease
AHCVD: acute hemorrhagic cerebrovascular disease
AUC: area under the curve
TH: thyroid hormones
ESS: euthyroid sick syndrome
TSH: thyroid-stimulating hormone
NIHSS: National Institutes of Health Stroke Scale
mACVD: mild ACVD
iACVD: intermediate ACVD
sACVD: severe ACVD
SBP: systolic blood pressure
DBP: diastolic blood pressure
CR: serum creatinine
BUN: blood urea nitrogen
ALT: alanine aminotransferase
AST: aspartate aminotransferase
TRH: thyrotropin-releasing hormone
CVD: cerebrovascular disease
TT3: total triiodothyronine
FT3: free triiodothyronine
TT4: total thyroxine
FT4: free thyroxine
CT: computed tomography
PC: primarily cured
SI: significantly improved
Im: improved
NC: no change
